# Effect of Rituximab on the cognitive impairment in patients with secondary progressive multiple sclerosis

**DOI:** 10.22088/cjim.13.3.484

**Published:** 2022

**Authors:** Saeideh Salehizadeh, Roghayyeh Saeedi, Mohammad Ali Sahraian, Hossein Rezaei Aliabadi, Seyedeh Nafiseh Hashemi, Sharareh Eskandarieh, Mohammad Reza Gheini, Shaghayegh Shahmirzaei, Mahsa Owji, Abdorreza Naser Moghadasi

**Affiliations:** 1 Multiple Sclerosis Research Center; Neuroscience institute; Tehran University of Medical Sciences; Tehran; Iran; 2 Bam University of Medical Sciences, Bam, Iran; 3 Statistics Department, Science Faculty, Shiraz University, Shiraz, Iran.; 4 Department of Neurology, Sina Hospital, Tehran University of Medical Sciences; Tehran; Iran

**Keywords:** Secondary Progressive Multiple Sclerosis (SPMS), Rituximab, MACFIMS، Cognitive impairment

## Abstract

**Background:**

The present study aimed to address the effect of Rituximab on the cognitive impairment in patients with secondary progressive MS (SPMS).

**Methods:**

The present interventional study used a convenience sampling method to select the study participants from SPMS patients. All these patients had progressive disability over the last two years before being admitted in the study. Prior to the administration of Rituximab, the minimal assessment of cognitive function in the multiple sclerosis (MACFIMS) test was performed for each patient who was a candidate to be included in this study. This test was repeated by passing 6 and 12 months from the initial treatment with Rituximab. Since the data needed for this study were obtained at different time intervals, so a linear mixed model was used for their analysis. Analysis of variance (ANOVA) was also used to investigate whether time and sex generally affect the cognitive impairments in SPMS patients. A p-value <0.05 was considered as statistically significant in this study.

**Results:**

Of the total 35 patients, 34% and 66% were men and women with a mean age of 41.33 and 41.39 years old, respectively. Rituximab showed a significant positive effect on a number of subgroups of MACFIMS test, including Controlled Oral Word Association Test (COWAT) (P-value: 0.038) and Brief Visuospatial Memory Test (BVMT-total) (P: 0.019).

**Conclusion:**

The present study revealed that Rituximab has a positive effect on the cognitive impairment resulted from MS in secondary progressive patients.

Multiple Sclerosis (MS) is a chronic inflammatory disease of the central nervous system (CNS), which can cause a wide range of symptoms and complaints. Accordingly, among them, the cognitive impairment can be regarded as one of the most important one. Cognitive impairment is a common problem among MS patients with a prevalence rate of 43-70% as reported in various studies (1, 2). This impairment can affect different cognitive aspects of the MS patient. Moreover, the most common type of cognitive impairment in MS patients is the information processing speed (IPS) impairment (3). The rate of the cognitive involvement varies among different types of MS. In general, it has been indicated that the rate and severity of the cognitive impairment both are higher in progressive forms of MS compared to the relapsing remitting forms (4). In a study conducted by Huijbregts et al., 108 relapsing remitting MS (RRMS) patients, 71 secondary progressive MS (SPMS) patients, 55 primary progressive MS (PPMS) patients, and 67 healthy controls were examined by performing neuropsychological tests. Correspondingly, this study revealed some significant findings regarding the differences in various types of MS in terms of the cognitive involvement.

The results of cognitive tests revealed that SPMS patients mostly indicate more impairments compared to PPMS patients. In contrast, it was shown that RRMS patients indicate a better cognitive performance in all tests compared to SPMS and PPMS patients (5). Therapeutically, symptomatic treatments unfortunately have a little positive effect on improving MS patients’ cognitive status (6); therefore, more attention must be paid to the mental rehabilitation to improve the symptoms (7). In addition, it should be noted that MS maintenance therapies ,including immunomodulators, can consequently improve patients’ cognitive status due to affecting the whole disease’s process (6, 7). However, it should be considered that most of these maintenance therapies could mainly affect RRMS patients. Therefore, it can be conjectured that the effects of these drugs on cognitive impairments might be limited only to these patients. In this case, it should not be expected to observe these drugs’ significant effect on the cognitive impairment of SPMS patients, for whom the duration of the disease is longer and the degeneration phase is more pronounced. Moreover, it is of great value to identify a new drug with a positive effect on the cognitive impairment in SPMS patients.

Rituximab is one of the relatively new maintenance therapies in the field of MS , which is increasingly used for the treatment of this disease (8). Rituximab has also been used for patients with SPMS in several previous studies (9-11). Considering the positive effect of this drug on secondary progressive patients, it is conceivable that this drug can also bring a positive effect on these patients’ cognitive impairment. So, the present study aimed to investigate the effect of Rituximab on the cognitive impairment in SPMS patients.

## Methods

Participants: The present interventional study used a convenience sampling method, to select the eligible participants from SPMS patients referred to the MS Clinic of Sina Hospital, Iran, from November, 2016 to November, 2017, in terms of the Lorscheider et al.’s criteria (12).

All the patients had progressive disability over the last two years before being admitted in the study. In addition, they were candidates for receiving Rituximab. Sustained progression was defined as having at least 1 point of Expanded Disability Status Scale (EDSS) during the three- to six-month follow-up, if the initial EDSS of patient was between 1-5 and 5. Notably, if the initial EDSS was more than 5.5, a 0.5-point increment during 3-6 months was considered as the sustained progression (12). None of the participants had received any corticosteroids over the last month prior to their admission to this study. Moreover, the patients with another concomitant disease that could affect the cognitive abilities such as cerebrovascular disease, learning disability, traumatic brain injury, hypothyroidism, and psychiatric diseases were excluded from this study (13). In addition, the patients who could not complete the one-year study period due to any reason, were excluded from this study, as well.

Evaluations: The patients’ demographic characteristics, including age, sex, duration of disease, duration of diagnosis, history of any previous disease, and EDSS score were recorded. Prior to the administration of Rituximab, MACFIMS test was performed by a clinical psychologist for each SPMS patient who was a candidate to be included in this study. This test was repeated by passing 6 and 12 months from the initial treatment with Rituximab. In the second and third administrations of this test, alternative MACFIMS tests were also used by the same clinical psychologist to prevent patients’ memorization of the answers due to the administration of repeated tests. In this regard, California Verbal Learning Test (CVLT), Brief Visuospatial Memory Test (BVMT), Delis-Kaplan Executive Function System (D_KEFS), Controlled Oral Word Association Test (COWAT), and Paced Auditory Serial Addition Test (PASAT) are alternative tests. The patients’ EDSS was also measured by a neurologist and re-recorded by passing 6 and 12 months from their initial treatment. All the patients were monitored by clinical visits every four months for finding any symptom of possible disease attacks during the one-year study period. All the included patients received 1g of Rituximab (Zytux) as the first injection. Accordingly, the mentioned dose was repeated on day 14. Thereafter, all the patients received 1g of Rituximab, as the maintenance drug, every 6 months (12). Zytux^TM^ is a biosimilar product, and the generic name Rituximab is a product of AryoGen Pharmed pharmaceutical company. A previous study addressed patients with chronic lymphocytic leukemia (CLL) and revealed that the efficacy and the rate of its side effects are equal to those of MabThera (13). In an observational study, the efficacy and safety of this drug in MS patients were investigated (12). The second and third MACFIMS tests were also performed the day after receiving the maintenance dose of Rituximab. Cognitive assessment: As mentioned earlier, MACFIMS test was used for the cognitive assessment. All the tests were done by an experienced clinical psychologist.The MACFIMS test is a set of the following seven different sub-tests: the Paced Auditory Serial Addition Test (PASAT), Symbol Digit Modalities Test (SDMT), Brief Visuospatial Memory Test-Revised (BVMT-R) for memory assessment, Controlled Oral Word Association Test (COWAT), Delis-Kaplan Executive Function System (D-KEFS) Sorting Test, California Verbal Learning Test Second Edition (CVLT-II), and Judgment of Line Orientation Test (JLO). Correspondingly, these are used to examine five parts of cognition, including processing speed, working memory, recent memory, spatial processing, and higher executive function (14). This test has also been translated into Farsi and then validated (15). MACFIMS is known as the most important test for the evaluation of different aspects of cognition in patients with MS. In the current study, the test was performed by a trained psychologist and then scored in terms of its manual. CVLT-II measures verbal learning and memory, and CVLT-II evaluates the delayed recall. In addition, PASAT assesses the information processing speed, attention, and calculation abilities. SDMT is a test used for the evaluation of sustained attention and processing speed. BVMT-R measures visuospatial learning and memory in the affected patients. JLO evaluates the visuospatial ability. COWAT is a test used for measuring verbal fluency, and D-KEFS is used for measuring executive functions in patients with MS. Data analysis: The collected data were entered into SPSS software, Version 22.0. Since the data needed for this study were obtained at different time intervals, so a linear mixed model was used for their analysis. Analysis of variance (ANOVA) was also used to investigate whether time and sex generally affect the cognitive impairments in SPMS patients. A p-value <0.05 was considered as statistically significant in this study. Ethical considerations: At first, the objectives of the study were thoroughly explained to the patients before the start of the study. Thereafter, a written consent was obtained from all the enrolled patients. The present study has been approved with the code of "IR.TUMS.MEDICINE.REC.1396.2973" by the Ethics Committee of Tehran University of Medical Sciences, Tehran, Iran.

## Results

At the beginning of the study, 40 patients were included; however, only 35 patients finally completed the study. Of the total 35 patients, 34% and 66% were men and women with a mean age of 41.33 and 41.39 years old, respectively. The mean duration of the disease for male and female subjects was 11.58 and 11.37 years, respectively (table 1). Furthermore, the mean scores of EDSS and MACFIMS tests in all three measurements are presented in table 2.

**Table 1:** The main characteristics of patients

	Mean N=35	Std. deviation
Age	41.37	8.55
Education *(years)*	12.49	3.10
Duration of disease	12.91	6.01
Duration of diagnosis EDSS	11.46 5.80	6.11 1.77
	**N**	**percent**
Sex *(female)*	23	65.7
Smoking	6	17.1
*Past Medical History *		
Hypertension	1	2.9
Coronary artery disease	3	8.6
Hyperthyroidism	3	8.6
Renal calculi	1	2.9
Testicular cancer	1	2.9
**None **	**26**	**74.3**

EDSS: Expanded Disability Status Scale

The results of the effect of Rituximab on each subset of the MACFIMS test are shown in table 3.

1) CVLT: Rituximab had no significant effect on MS patients’ cognitive impairment, which was assessed by CVLT test over time (P-value: 0.65). In addition, it did not yield any difference between male and female patients. Moreover, no decline was observed in this cognitive aspect over time.

2) JLO: Rituximab had no significant effect on MS patients’ cognitive impairment, which was assessed by JLO test over time (P-value: 0.96). In addition, it did not yield any difference between male and female patients. Moreover, no decline was observed in this cognitive aspect over time.

3) COWAT: Rituximab had a significant effect on MS patients’ cognitive impairment, which was assessed by COWAT test over time (P-value: 0.038); however, it did not yield any difference between male and female patients.

4) CVLT_delay: Rituximab had no significant effect on MS patients’ cognitive impairment, which was assessed by CVLT_delay test over time (P: 0.88). In addition, it did not yield any difference between male and female patients. Moreover, no decline was observed in this cognitive aspect over time.

5) BVMT_total: Rituximab had a significant effect on MS patients’ cognitive impairment, which was assessed by BVMT_total test over time (P: 0.019); however, it did not yield any difference between male and female patients.

6) SDMT: This test’s results indicated a significant improvement in the second round of testing compared to the results of the first round (P: 0.017); however, this effect was not observed in the third round of testing (P:0.37). Accordingly, this result was similar in both sexes.

7) PASAT: Rituximab had no significant effect on MS patients’ cognitive impairment, which was assessed by PASAT test over time (0.47). In addition, it did not yield any difference between male and female patients. Moreover, no decline was observed in terms of this cognitive aspect over time.

8) D-KEFS-description: This test’s results indicated a significant improvement in the second round of testing compared to the results of the first round (P- value: <0.0001); however, this effect was not observed in the third round of testing (0.058). Correspondingly, this result was similar in both sexes.

9) D-KEFS-sorting: This test’s results indicated a significant improvement in the third round of testing compared to the results of the first round (P<0.0001); however, this effect was not observed in the second round of testing. As well, this result was similar in both sexes.

10) BVMT_delay: This test’s results indicated a significant improvement in the second round of testing compared to the results of the first round (P:0.034); however, this effect was not observed in the third round of testing (P: 0.30). Accordingly, this result was similar in both sexes.

**Table 2:** Mean and SD for the different parameters of cognitive assessment and EDSS in three times

*P-value*	Time3	*P-value*	Time2		Time1	variables
SD	Mean	SD	Mean	SD	Mean	
**11.74 0.80**	48.43	10.91 0.43	46.03	10.25	40.86	CVLT
**17.82 0.21**	22.5	4.85 0.18	19.33	5.32	18.79	JLO
**12.07 0.02**	23.37	9.55 0.53	20.66	10.25	20.86	COWAT
**4.0 0.85**	10	3.0 0.92	9.0	3.0	9.0	CVLT_delay
**8.85 0.01**	20.83	8.76 0.04	19.43	8.04	18.4	BVMT_total
**12.8 ** **0.37**	36.09	13.74 0.017	37.09	13.24	34.89	SDMT
**8.67 0.74**	51.04	10.15 0.38	50	8.02	47.79	PASAT
**10.37 ** **0.058**	15.62	9.53 <0.0001	10.21	11.32	21.12	D-kefs-description
**12.16 0.08**	19.44	3.7 <0.0001	4.97	2.93	5.82	D-kefs-sorting
**3.60 ** **0.30**	8.08	4.50 0.034	8.4	3.3	7.4	BVMT_delay
**1.44 0.65**	5.76	1.36 0.81	5.83	1.77	5.80	EDSS

BVMT-Delay: Brief Visuospatial Memory Test-Delay; BVMT-total: Brief Visuospatial Memory Test-total; COWAT: Controlled Oral Word Association Test; CVLT: California Verbal Learning Test; CVLT_delay: California Verbal Learning Test_delay ; D_KEFS_sorting: Delis-Kaplan Executive Function System; EDSS: Expanded Disability Status Scale; JLO: Judgment of Line Orientation Test; PASAT: Paced Auditory Serial Addition Test; SDMT: Symbol Digit Modalities Test

**Table 3:** Mixed model regression results for variables of the test

	*1 (month 0)*	*2 (month 6)*	3 (month 12)	P-value	P-value Sex*time
CVLT	40.86	46.03	48.43	0.65	0.36
CVLT_delay	9.0	9.0	10	0.88	0.19
SDMT	34.89	37.09	36.09	0.37	0.20
BVMT-total	18.4	19.43	20.83	0.019	0.11
BVMT-Delay	7.4	8.4	8.08	0.30	0.45
COWAT	20.86	20.66	23.37	0.038	0.38
D_KEFS_sorting	5.82	4.97	19.44	0.0001	0.82
D_KEFS_description	21.12	10.21	15.62	0.058	0.84
JLO	18.79	19.33	22.5	0.96	0.70
**PASAT**	**47.79**	**50**	**51.04**	**0.47**	**0.55**

BVMT-Delay: Brief Visuospatial Memory Test-Delay; BVMT-total: Brief Visuospatial Memory Test-total; COWAT: Controlled Oral Word Association Test; CVLT: California Verbal Learning Test; CVLT_delay: California Verbal Learning Test_delay ; D_KEFS_sorting: Delis-Kaplan Executive Function System; JLO: Judgment of Line Orientation Test; PASAT: Paced Auditory Serial Addition Test; SDMT: Symbol Digit Modalities Test

## Discussion

The findings of the present study revealed the effects of Rituximab on the cognitive impairment in SPMS patients. Although this study had no control group, a decrease was expected in some aspects of the patients’ cognition in terms of the course of the cognitive impairment caused by MS disease. Previous studies have indicated that the affected patients mostly experience a decline in some aspects of their cognition over time (14, 15). However, this decline was not observed following the administration of Rituximab. It should be noted that some degrees of improvement were observed in some cognitive aspects.

The effect of maintenance drugs on cognitive impairments in MS patients has been previously demonstrated in some studies. According to a pioneer study in this regard, an early initiation of IFNβ-1b has a positive and persistent effect on the PASAT test (16). The study by Barak and Achiron also showed the same effect of beta interferon on MS patients’ cognitive impairment (17). As well, a similar effect has been observed following using both Fingolimod (18) and Natalizumab (19). According to the results of the de Flon et al.’s study, Rituximab had positive effects on treatment satisfaction and cognitive symptoms improvement in Relapsing Remitting Multiple Sclrosis (RRMS) during a 2-year follow-up (20). The effect of maintenance drugs on the cognitive impairment was shown to be due to the overall effect of these drugs on the course of MS. In fact, the positive effects of these drugs on the brain as well as on the prevention of MS progression consequently cause a positive effect on the cognitive impairment of these patients. Therefore, the positive effect of Rituximab should follow the same mechanism and explanation. Rituximab is a chimeric monoclonal antibody against CD 20 B-cells, which has been revealed to be effective on the treatment of central nervous system disorders (1, 5, 9, 21). Numerous studies have previously reported the effect of Rituximab on SPMS. In this regard, a systemic review conducted by Trivino et al. revealed the positive effect of this drug on patients with relapsing and progressive types of MS (9). The results of a study conducted by Perrone et al. in 2014 (22) on SPMS patients also showed that Rituximab could be effective on stabilizing the progression of the disease course and it could also reduce the disease progression potentially. Moreover, Sulzer et al. conducted a retrospective multi-center study in 2016, which revealed the positive effect of Rituximab on various types of MS, including SPMS (24). In a study performed by Alldredge et al., the positive effect of Rituximab on patients with progressive MS was determined (11).Additionally, another study by Naegelin in 2019 reported that patients with SPMS treated with Rituximab had lower EDSS scores than the controls (23).

As the above-mentioned studies have revealed, Rituximab can have a positive effect on SPMS patients. In our study, none of the included patients had an attack. On the other hand, their disease reached stability and did not progress anymore. The mentioned findings indicated the positive effect of the drug in terms of the progression of disability on the patients studied in this research. Hence, the lack of progress in the cognitive impairment as well as improvements in its significant aspects can be justified by considering the effect of Rituximab on the target patients. In addition, lack of progression may consequently affect the psychological status of patients and also improve their cognitive abilities.

This study had some limitations and drawbacks that should be addressed in future studies. Firstly, this study had no control group. Secondly, MRI and detailed imaging were not used to evaluate the effects of the drug on neural pathways. In this regard, the prolonged follow-up of these patients can also help to improve the study and result in new findings. Moreover, considering the psychological aspects of SPMS as well as the evaluation of the effect of Rituximab can be regarded as the strengths of this study. Whether this drug has a direct effect on cognition or not, it is an important issue for further studies. The present study revealed that Rituximab has a positive effect on the cognitive impairment caused by MS in secondary progressive patients. It was shown that the effects of this drug are probably related to its overall effects on this particular group of patients.
